# Multilocus Sequence Typing of *Borrelia burgdorferi* Suggests Existence of Lineages with Differential Pathogenic Properties in Humans

**DOI:** 10.1371/journal.pone.0073066

**Published:** 2013-09-17

**Authors:** Klara Hanincova, Priyanka Mukherjee, Nicholas H. Ogden, Gabriele Margos, Gary P. Wormser, Kurt D. Reed, Jennifer K. Meece, Mary F. Vandermause, Ira Schwartz

**Affiliations:** 1 Department of Microbiology and Immunology, New York Medical College, Valhalla, New York, United States of America; 2 Zoonoses Division, Centre for Food-borne, Environmental and Zoonotic Infectious Diseases, Public Health Agency of Canada, Ottawa, Ontario, Canada; 3 Institute for Infectious Diseases and Zoonoses, Ludwig-Maximilians-University Munich and National Reference Centre for Borrelia at the Bavarian Health and Food Safety Authority, Oberschleissheim, Germany; 4 Division of Infectious Diseases, Department of Medicine, New York Medical College, Valhalla, New York, United States of America; 5 Department of Pathology and Laboratory Medicine, University of Wisconsin, Madison, Wisconsin, United States of America; 6 Marshfield Clinic Research Foundation, Marshfield, Wisconsin, United States of America; University of Kentucky College of Medicine, United States of America

## Abstract

The clinical manifestations of Lyme disease, caused by *Borrelia burgdorferi,* vary considerably in different patients, possibly due to infection by strains with varying pathogenicity. Both rRNA intergenic spacer and *ospC* typing methods have proven to be useful tools for categorizing *B. burgdorferi* strains that vary in their tendency to disseminate in humans. Neither method, however, is suitable for inferring intraspecific relationships among strains that are important for understanding the evolution of pathogenicity and the geographic spread of disease. In this study, multilocus sequence typing (MLST) was employed to investigate the population structure of *B. burgdorferi* recovered from human Lyme disease patients. A total of 146 clinical isolates from patients in New York and Wisconsin were divided into 53 sequence types (STs). A goeBURST analysis, that also included previously published STs from the northeastern and upper Midwestern US and adjoining areas of Canada, identified 11 major and 3 minor clonal complexes, as well as 14 singletons. The data revealed that patients from New York and Wisconsin were infected with two distinct, but genetically and phylogenetically closely related, populations of *B. burgdorferi*. Importantly, the data suggest the existence of *B. burgdorferi* lineages with differential capabilities for dissemination in humans. Interestingly, the data also indicate that MLST is better able to predict the outcome of localized or disseminated infection than is *ospC* typing.

## Introduction

Lyme disease is a multisystem illness that, in North America, is caused by the spirochete *Borrelia burgdorferi* sensu stricto (hereafter referred to as *B. burgdorferi*) which is transmitted to humans through the bite of infected *Ixodes* spp. ticks [1]. In the United States, Lyme disease remains the leading cause of all vector-borne human infections with more than 20,000 annually reported cases [2]. The risk of infection is highly localized within 12 states in the northeastern and upper Midwestern regions accounting for 94% of all reported cases [2]. Clinical features of human infection can include a wide variety of symptoms ranging from a characteristic skin lesion known as erythema migrans often seen during the early stages of disease to more severe musculoskeletal, neurologic or cardiovascular manifestations of disseminated infection that arise from hematogenous dissemination from the initial site of inoculation in the skin [3], [4].

Substantial genetic diversity exists within *B. burgdorferi* [5]–[13]. The plasmid-borne, highly polymorphic outer surface protein C gene (*ospC*) and the 16 S–23 S (*rrs-rrl*) rRNA intergenic spacer (IGS) have been the most commonly used genetic markers for *B. burgdorferi* strain identification in the US [6], [10], [12]–[17]. It has been observed that strains exhibiting restriction fragment length polymorphism in the 16 S–23 S rRNA intergenic spacer designated as RST1 or possessing *ospC* major groups A, B, H, I and K have a stronger tendency for hematogenous dissemination early in the course of disease [14], [16], [18]–[22]. This observation gave rise to the concept that a distinct subset of *B. burgdorferi* genotypes is responsible for early disseminated infection in humans, suggesting that some degree of differential pathogenicity exists among strains. Both RST and *ospC* typing methods provide a useful tool for categorizing *B. burgdorferi* strains that vary in their tendency to disseminate in humans. Neither method, however, is suitable for inferring intraspecific relationships among strains that are important for understanding the evolution of pathogenicity and the geographical spread of disease. While RST typing has limited discriminatory power for this purpose [13], [23] the suitability of *ospC* typing may also be restricted since the highly variable *ospC* gene is subject to recombination and horizontal gene transfer, as well as strong selection by the host immune system [7], [8], [24]–[28]. Moreover, phylogenetic analysis of a single locus can often result in erroneous inference of evolutionary relationships [29], [30].

The most appropriate of the current techniques for large-scale epidemiology, strain identification and understanding of the population structure of bacterial species is multilocus sequence typing (MLST). This method is based on nucleotide sequences of multiple housekeeping genes that are evolving nearly neutrally. MLST analysis has been used successfully to study a number of bacteria (http://www.mlst.net and http://www.pubmlst.org) and has been employed to identify lineages of particular clinical relevance in bacterial pathogens such as *Neisseria meningitidis* [29], [31], *Streptococcus pneumoniae* [32], [33], *Staphylococcus aureus* [34]–[36], *Campylobacter jejuni* [37]–[39] and *Bartonella henselae* [40]. An MLST method based on eight housekeeping loci providing a high degree of intraspecies discriminatory power has been recently developed and validated for *B. burgdorferi* [7]. This MLST typing scheme has been employed to examine the population structure of *B. burgdorferi* in *Ixodes scapularis* and *Ixodes pacificus*, the principal vectors of Lyme disease in North America [7], [41]–[43]. To date, however, MLST has not been used to assess the population structure of clinically relevant strains of *B. burgdorferi*.

In this study, MLST was used for the first time to explore the population structure of *B. burgdorferi* isolated from Lyme disease patients. The genetic diversity of clinical isolates was assessed, and the genetic and evolutionary relationships between strains found in patients with localized versus disseminated infection, and in patients from two different geographical locations in the US, New York and Wisconsin, were evaluated. The data suggest the existence of *B. burgdorferi* lineages with differential pathogenic properties in humans.

## Results

### MLST and Identification of Clonal Complexes

**Figure 1:**
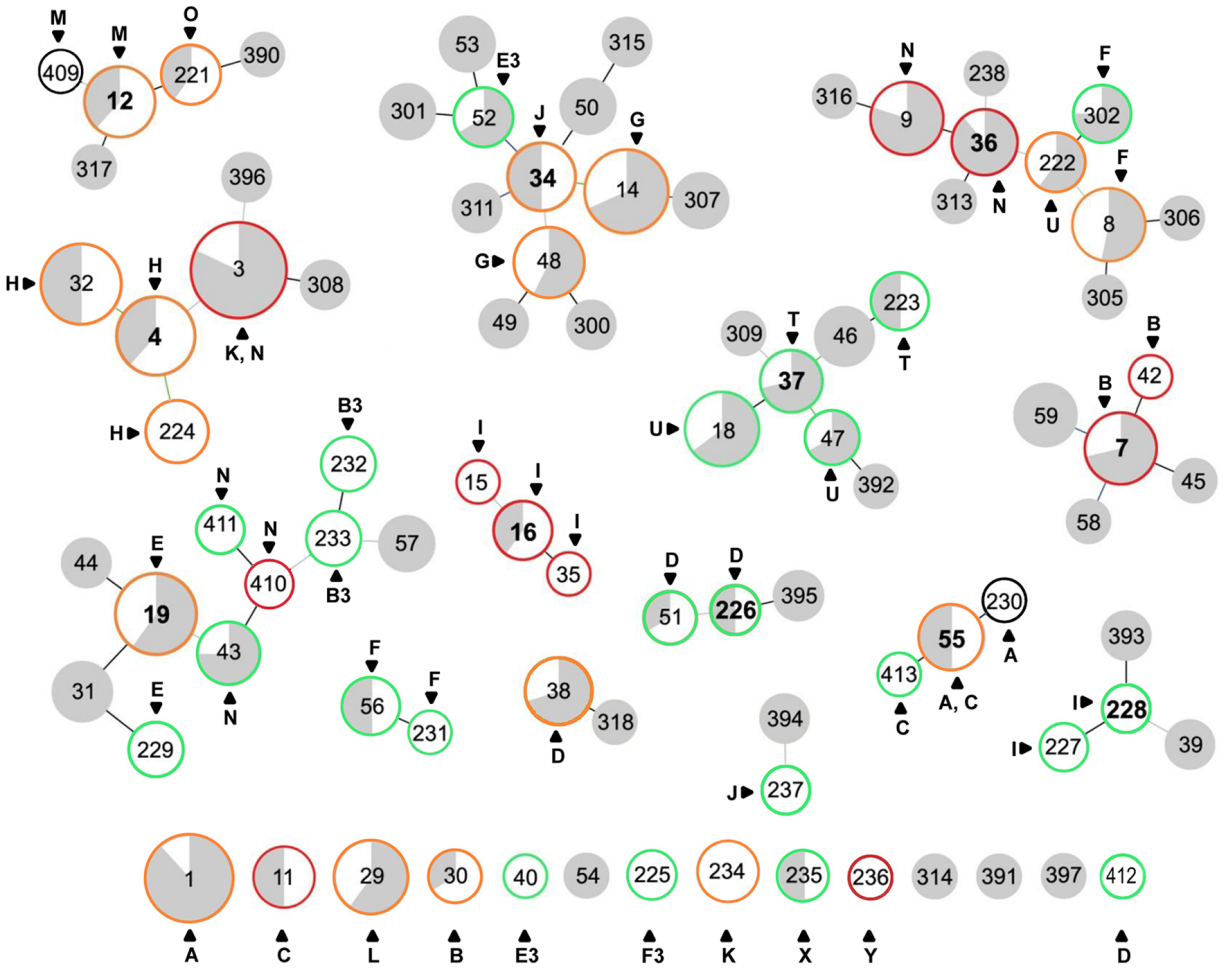
A population snapshot of *B. burgdorferi* in the northeastern and midwestern United States and Canada. The snapshot comprises of 88(420 human and tick samples) and was created by goeBURST v1.2 using data from this study and the previously published data sets downloaded from http://borrelia.mlst.net/ [7], [41]–[43]. Circle size and color correspond to MLST sample size and the source, respectively. Gray, strains found in ticks; white, strains found in humans. Colored lines connecting STs indicate descending order of certainty; black lines are inferred without tiebreak rules, blue lines are inferred using tiebreak rule 1 (number of SLV), and green lines are inferred using tiebreak rule 2 (number of DLV). STs connected by a black line are single locus variants and STs connected by gray line are double locus variants. The inferred founders of clonal complexes are numbered in bold. STs found in patients with localized infection are outlined in green, those found in patients with disseminated infection are outlined in red, those found in both patients with localized and patients with disseminated infection are outlined in orange and those found in patients with undetermined clinical status are outlined in black. STs that are not outlined were only found in ticks. The *ospC* major groups are shown for all 53 STs which were found in humans.

**Figure 2:**
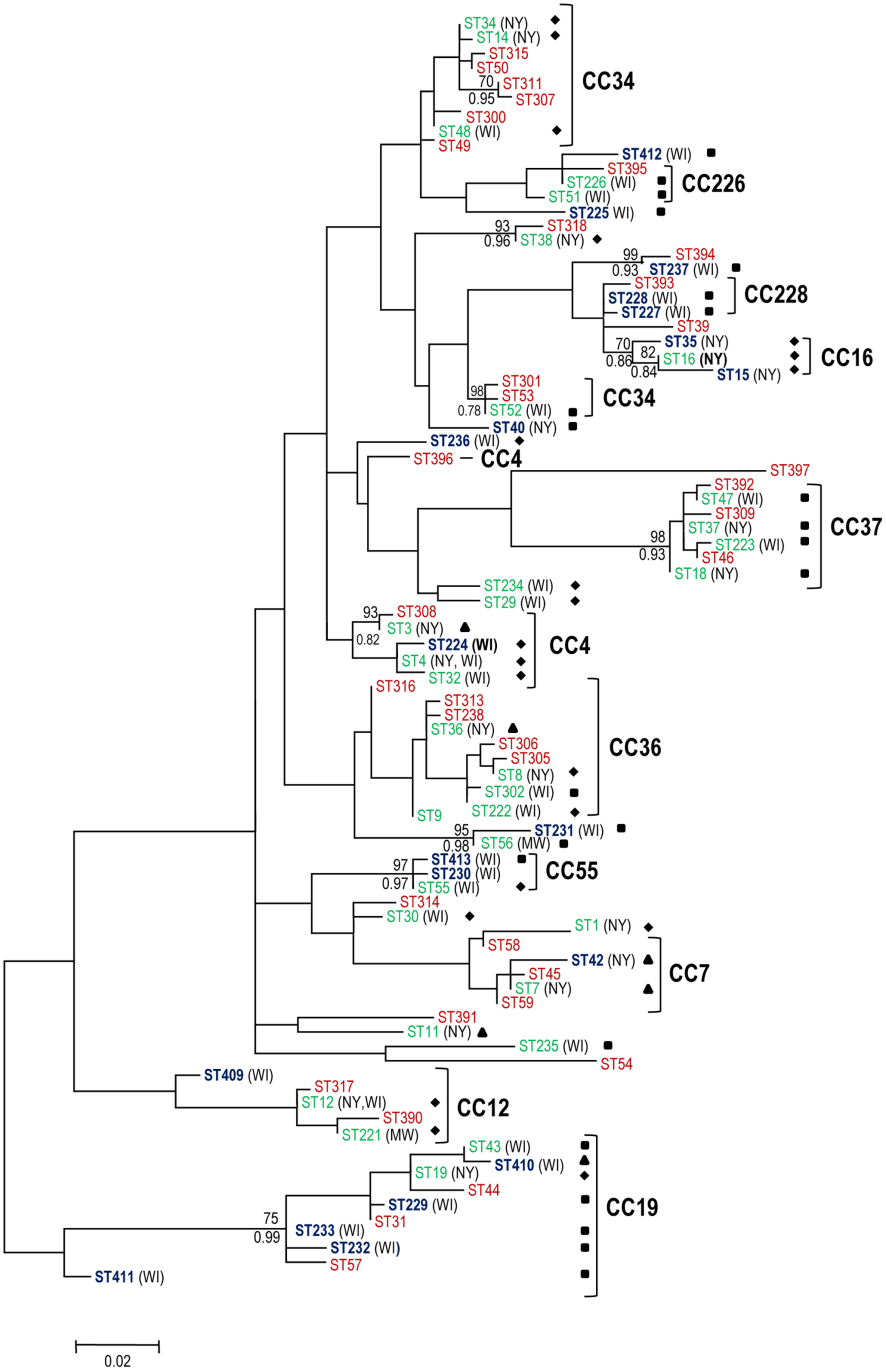
Unrooted ML tree of *B. burgdorferi* based on concatenated sequences of eight MLST housekeeping genes. The tree was created using data from this study and the previously published data sets downloaded from http://borrelia.mlst.net/ [7], [41]–[43]. A total of 420 *B. burgdorferi* samples (88 STs) found in humans and ticks from the northeastern United States and Canada were used. The aLRT statistical values and nonparametric bootstrap values for highly supported nodes in both maximum parsimony (with >70% support) and maximum likelihood (with aLRT >0.9 support) are indicated above and below the branches, respectively. STs newly identified in this study are in bold. The grouping of STs into major clonal complexes (CCs) is indicated by right brackets. The STs found only in humans are shown in blue, those found only in ticks are shown in red and those found in both humans and ticks are shown in green. The type of infection is indicated next to the ST using solid square (ST found in patients with localized infection), solid triangle (ST found in patients with disseminated infection) and solid diamond (ST found in both patients with localized and patients with disseminated infection). Geographical origin of STs found in humans and identified in this study is indicated in brackets next to the STs (NY – New York; WI – Wisconsin).

MLST analysis of 146 *B. burgdorferi* isolates recovered from Lyme disease patients in New York and Wisconsin revealed 53 sequence types (STs) (Table S1); 23 have been previously identified and reported [7], [41]–[43]. Twenty-two of the 53 STs were represented by only a single isolate, while the number of isolates belonging to other STs ranged from 2 to 8 (Table S1). A goeBURST analysis, performed including previously published STs from the northeastern and upper midwestern US and adjoining areas of Canada, grouped the new STs into eleven major clonal complexes, five of which were new (CC4, CC16, CC226, CC55 and CC228) and six of which (CC7, CC12, CC19, CC34, CC36 and CC37) were described previously [7], [41]–[43]. Three minor clonal complexes and 14 singletons were also identified (Figure 1). Two of the eleven major clonal complexes (CC37 and CC55) were clearly characterized as highly supported terminal clusters on a phylogenetic tree constructed from concatenated sequences of the eight MLST genes (Figure 2). CC36, CC12, CC16 and CC19 also formed clusters on the tree, but did not attain sufficient statistical support (>70% on the MP tree and aLRT >0.9 on the ML tree). CC4 and CC34 included STs that were placed outside their main cluster on the tree (Figure 2). Further examination of ST396 (an outlier in CC4) and ST4 (the inferred founder of CC4) revealed that three variant loci (*clpA*, *pyrG* and *uvrA*) differed at multiple sites (6, 2 and 3, respectively), resulting in a total of 11 polymorphisms, which on the tree led to the separation of ST396 from the other STs assigned to CC4. Similarly, the comparison of ST52 (an outlier in CC34) and ST34 (the inferred founder of CC34) revealed seven polymorphisms in the *clpA* locus, which resulted in the separation of ST52 (and its descendants) from the other STs assigned to CC34.

### Relationship between *ospC* Major Groups and STs

For 29 of 31 STs that contained ≥2 isolates there was a perfect congruence between ST and *ospC* major group. In the other two STs (ST3 and ST55) a single isolate possessed an *ospC* major group different from the other isolates (Table S1 and Figure 1). However, closely related STs did not always exhibit the same *ospC* major group. Seven out of 11 major clonal complexes (CC4, CC12, CC19, CC34, CC36, CC37 and CC55) contained STs of different *ospC* major groups (Figure 1). Furthermore, only three (L, O and F3) out of 20 *ospC* major groups that contained ≥2 isolates were restricted to a single ST; the remaining *ospC* major groups were associated with multiple STs. The *ospC* major groups distributed among the largest number of STs were N (6 STs), I (5 STs) and F (4 STs). In addition, *ospC* major groups F, I, N and U were shared by isolates of different major clonal complexes (Table S1 and Figure 1). By definition, *ospC* major groups can contain closely related alleles that differ from each other in less than 2% of their nucleotide sequence [12]. To assess whether the *ospC* sequences would allow further differentiation between STs and major clonal complexes, we compared sequences of isolates within 17 *ospC* major groups that were associated with multiple STs. Nine *ospC* major groups, including N and I, that were associated with the greatest number of STs were represented by a single *ospC* allele. *ospC* major groups D, F, H and M contained two or more *ospC* alleles, some of which were shared by multiple STs. However, with exception of *ospC* major group M, isolates belonging to the same ST possessed identical *ospC* alleles. *ospC* major groups C, E, U and E3 were each represented by two *ospC* alleles, each of which were associated with different STs, but some of these STs were represented by only one isolate (Table S1).

### Geographical Distribution of STs and *ospC* Major Groups Found in Lyme Disease Patients

**Figure 3:**
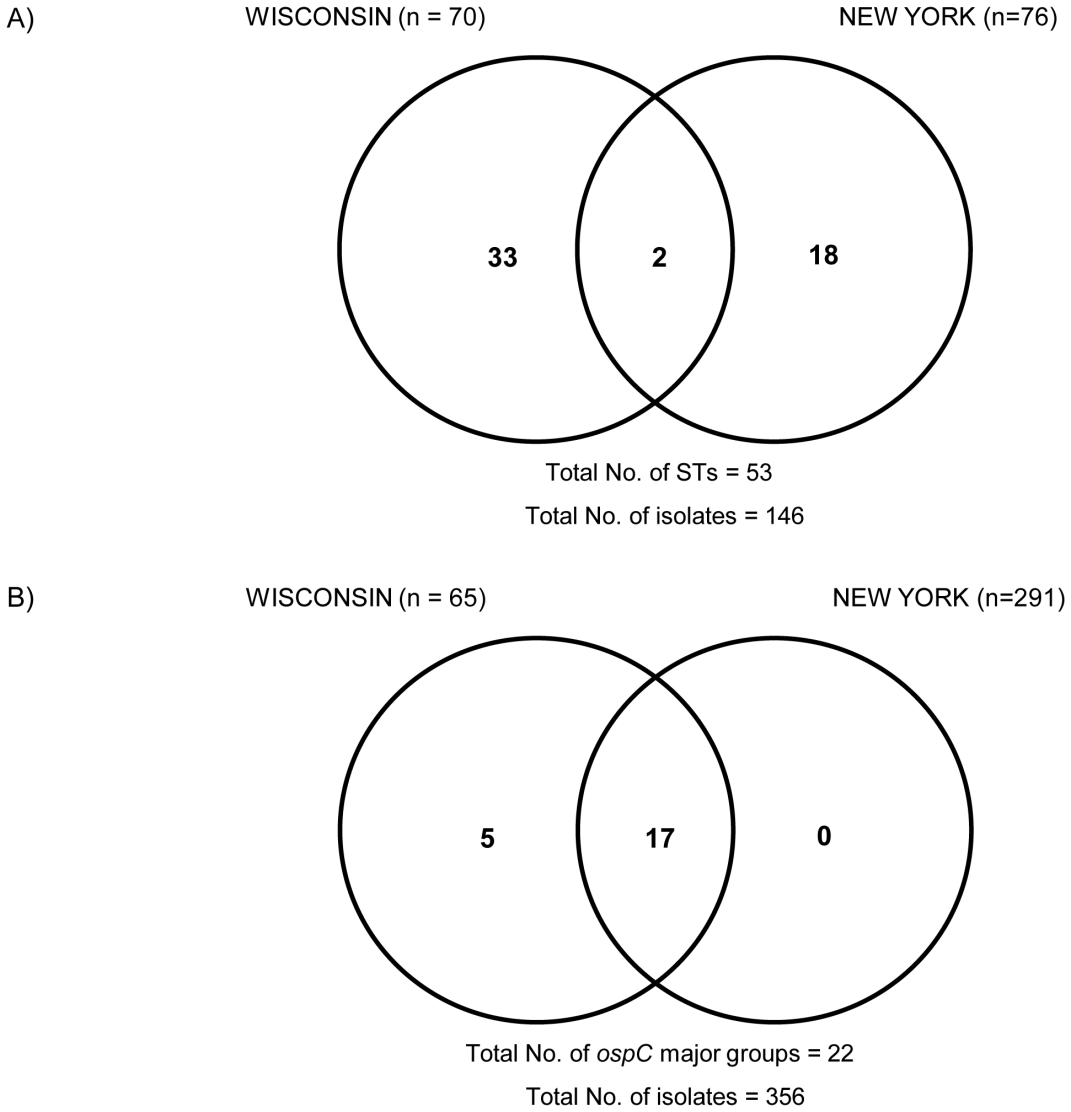
Venn diagrams depicting the geographical distribution of *B. burgdorferi* in Lyme disease patients. A) Geographical distribution of *B. burgdorferi* STs found in Lyme disease patients from New York and Wisconsin. B) Geographical distribution of *B. burgdorferi ospC* major groups identified in skin samples of Lyme disease patients from New York and Wisconsin.

Out of 146 patients, 76 were from New York and 70 from Wisconsin. Patients from Wisconsin appeared to have a higher ST diversity (n = 35) than patients from New York (n = 20). Only two STs (ST4 and ST12) were found in both geographical locations (Table S1 and Figure 3A). Despite the almost entirely non-overlapping ST distribution (Figure 3A), the STs from New York patients clustered together with STs from Wisconsin patients on well-supported nodes in the ML tree. For example, ST18 and ST37 found only in patients from New York formed a strongly supported cluster with ST47 and ST223 found only in patients from Wisconsin (Figure 2). Furthermore, the genetic relatedness of the two populations was corroborated by a permutation test using the allelic profiles of these isolates (multiple response permutation procedure, P>0.05). In addition, all clonal complexes consisted of STs from both geographical locations.

The diversity and frequency distribution of *ospC* major groups in Wisconsin patients (65 skin isolates cultured during 1995 to 2003) was compared with 291 previously genotyped skin isolates obtained from New York patients during 1991 to 2005 [13], [19] (Table 1). A greater diversity of *ospC* major groups was observed in skin isolates from Wisconsin (n = 22) than those from New York (n = 17) (Table 1). While all *ospC* major groups found in skin isolates from New York were also found in skin isolates from Wisconsin, *ospC* major groups L, X, Y, B3 and F3 were found only in skin isolates from Wisconsin (Table 1, Figure 3B). The frequency distribution of *ospC* major groups differed significantly between skin isolates from New York and Wisconsin (correspondence analysis, χ2 = 119.88, P<0.001, Table S2). The most common *ospC* major groups found in the skin isolates from New York were K, A, B and I which constituted 64.9% of all infections. In contrast, the most common *ospC* major group found in the skin isolates from Wisconsin was H, which constituted 18.5% of all infections.

**Table 1:** Geographical distribution of *B. burgdorferi ospC* major groups found in skin of Lyme disease patients from New York and Wisconsin.

	*ospC* major groups^b^																								
Location	A	B	C	D	E	F	G	H	I	J	K	L	M	N	O	T	U	X	Y	B3	E3	F3	TOTAL		
New York^a^	46	37	2	4	14	9	14	13	20	3	86	0	11	17	1	2	11	0	0	0	1	0	291		
Wisconsin	2	2	2	4	2	4	3	12	2	1	4	5	2	3	3	2	3	1	1	4	1	2	65		
Total	48	39	4	8	16	13	17	25	22	4	90	5	13	20	4	4	14	1	1	4	2	2	356		

a New York data (n = 290) based on [19]. One additional isolate (E3) was added from [13].

b *ospC* major group designation according to [8], [12]. *ospC* major groups X and Y were not published at the time this article was written but are available in GenBank under accession numbers HM047876 and HM047875 respectively.

### Characteristics of Clinical Isolates, STs and Clonal Complexes

Data with regard to disseminated and localized infection were available for 127 isolates (Table S1). These isolates were divided by MLST into 51 STs. Of these, 41 linked to eleven major and three minor clonal complexes, and 10 were identified as singletons (Table 2 and Figure 1). Out of the 27 STs that were represented by ≥2 isolates with available clinical data, we found 17 in patients with both disseminated and localized infection (Table 3). While the association between type of infection and ST bordered on significant (total inertia = 0.522, χ^2^ = 65.84, P = 0.066), there was a significant association between type of infection and clonal complexes in each of three separate statistical analyses. The first analysis included major and minor clonal complexes as well as singletons (total inertia = 0.369, χ^2^ = 46.55, P = 0.003); in the second analysis, singletons were excluded (total inertia = 0.349, χ^2^ = 42.61, P = 0.001); and for the third analysis only major clonal complexes were included (total inertia = 0.329, χ^2^ = 33.60, P = 0.001) (Table 2 and Figure S1). In each scenario CC37 was clearly associated with localized infection and CC4 showed a tendency to include mainly isolates from patients with disseminated infections. CC7 and CC16 were also associated with disseminated infection, although the number of isolates within these clonal complexes was too small to draw any definite conclusions (Table 2 and Figure S1). *ospC* major groups were also significantly associated with type of infection, but in each case using the full data, and reduced data as described above, *ospC* groups appeared less predictive than clonal complexes; *ospC*-based analyses had lower inertia and χ^2^ values and/or higher P values (inertia = 0.303, 0.281, 0.336; χ^2^ = 38.14, 34.34, 34.29; and P = 0.012, 0.017 and 0.008, respectively, for the three analyses). *ospC* major groups B, H and K were particularly associated with disseminated infection and *ospC* major groups F and T were significantly associated with localized infection (Figure S1). Further, MLST allowed additional discrimination between isolates with differential disseminative properties that belonged to a single *ospC* major group. For example, all *ospC* major group U isolates in CC37 were cultured from patients with localized infection, whereas the only *ospC* major group U isolate that was cultured from a patient with a disseminated infection was a member of a different clonal complex (CC36). Similarly, *ospC* major group K isolates in CC4 were obtained from patients with disseminated infection; two *ospC* major group K isolates cultured from patients with localized infection were not members of CC4. An *ospC* major group B isolate from a patient with localized infection was not part of CC7, a clonal complex that is comprised only of *ospC* major group B isolates from patients with disseminated infection. *ospC* major group I isolates were divided between two different major clonal complexes. CC16 contains *ospC* major group I isolates cultured from patients with disseminated infection and CC228 is comprised of *ospC* major group I isolates cultured from patients with localized skin infection (Figure 1, Table S1). The discriminatory power of *ospC* did not improve much by considering the specific *ospC* sequence, as all isolates within *ospC* major groups B, I and K had identical *ospC* sequences.

**Table 2:** Distribution of *B. burgdorferi* isolates from Lyme disease patients with localized and disseminated infection between clonal complexes and singletons.

	Number of Isolates		
Clonal Complex/Singleton	Localized Infection	Disseminated Infection	Total
**CC4**	6	18	24
**CC7**	0	4	4
**CC12**	5	6	11
**CC16**	0	3	3
**CC19**	9	4	13
**CC34**	7	4	11
**CC36**	6	6	12
**CC37**	11	0	11
**CC55**	2	2	4
**CC226**	3	0	3
**CC228**	2	0	2
**CC38–318**	1	1	2
**CC56–231**	2	0	2
**CC237–394**	1	0	1
**S1**	1	4	5
**S11**	0	3	3
**S29**	1	3	4
**S30**	1	1	2
**S40**	1	0	1
**S225**	2	0	2
**S234**	3	1	4
**S235**	1	0	1
**S236**	0	1	1
**S412**	1	0	1
**Total**	66	61	127

**Table 3:** Distribution of *B. burgdorferi* STs between Lyme disease patients with disseminated and localized infection.

		STs^a^																										
Type of infection	1	3	4	7	8	9	11	12	14	18		19	29	30	32	34	37	38	48	51	55	221	222	223	224	225	233	234
**Localized**	1	0	1	0	4	0	0	4	4	6	3		1	1	4	1	2	1	1	2	1	1	1	2	1	2	2	2
**Disseminated**	4	4	7	3	1	3	3	4	1	0	3		3	1	4	2	0	1	1	0	2	2	1	0	3	0	0	1
**Total**	5	4	8	3	5	3	3	8	5	6	6		4	2	8	3	2	2	2	2	3	3	2	2	4	2	2	3

a Only STs that are represented by ≥2 isolates with available clinical data are shown.

STs, sequence types.

## Discussion

Accurate strain identification of pathogenic bacteria is essential for epidemiological surveillance and permits effective public health decisions. Previously, MLST based on eight housekeeping loci was exploited to examine *B. burgdorferi* population structure in *Ixodes* spp. ticks and provided novel insights into the phylogeography of the pathogen [7], [41]–[43]. Moreover, MLST has been shown to detect ecologically distinct groups within *B. garinii* (i.e., a bird-associated ecotype and a rodent-associated ecotype) [44] indicating that this typing method has the power to detect and demarcate phenotypic differences of ecological significance among Lyme disease spirochetes. The present study represents the first application of this MLST scheme for analysis of the *B. burgdorferi* population structure in patients with Lyme disease, and the results indicate that phylogeny and pathogenicity of *B. burgdorferi* are correlated.

Numerous *B. burgdorferi* genotypes have been previously characterized among isolates obtained from Lyme disease patients in the northeastern US, primarily using the *rrs-rrlA* IGS and *ospC* loci [13], [19]–[21], [45]–[47]. An association of certain *rrs-rrlA* and *ospC* genotypes with early dissemination has been observed [16], [18]–[20], [22], but the specific determinants facilitating dissemination remain unknown. The differences in disease severity and dissemination properties between different *B. burgdorferi* genotypes have been experimentally corroborated for selected strains in C3H/HeJ mice [48], [49]. The MLST analysis confirmed the high degree of genetic heterogeneity of *B. burgdorferi* in Lyme disease patients indicating that a wide range of *B. burgdorferi* strains is capable of infecting humans. Clinical isolates were represented in all the identified clonal complexes suggesting that there is no link between MLST genotype and the propensity to cause human infection *per se*. However, there was an association of certain clonal complexes with the type of infection (i.e., disseminated vs. localized) suggesting that some clonal complexes of *B. burgdorferi* may have a greater propensity for hematogenous dissemination, while others, such as members of CC37, may be capable of only causing localized skin infection. Analysis of the population structure using much larger data sets, in combination with careful epidemiological sampling, is required to better understand the relationship between dissemination potential and clonal complex.

Interestingly, the data indicate that the power of MLST to predict the outcome of infection (i.e., localized or disseminated) was greater than that of *ospC* typing, suggesting that the pathogenic traits of *B. burgdorferi* correlate with the phylogenetic signal. Thus, association between *ospC* major groups and dissemination properties may be indirect and operate via strong linkage disequilibrium [5], [6], [8], [13], [47]. Indeed, studies in mice have shown that OspC is a factor essential for initial establishment of infection in mammals, but it is also an effective immune target that must be down-regulated after the initiation of infection so as to prevent spirochete clearance [25], [50]. The ability of *B. burgdorferi* strains to cause disseminated infection in humans is a phenotypic characteristic of unknown origin. It is likely that *B. burgdorferi* interactions with its vectors and natural reservoir hosts throughout a long evolutionary course resulted in genotypes which in the human body display either disseminative or non-disseminative properties. Therefore, further studies of the evolutionary origins of different clonal complexes may help to predict the spatial and temporal occurrence of pathogenic *B. burgdorferi* strains.

Previously, *ospC* major groups A, B, H, I and K, isolated from patients in New York, have been identified predominantly as invasive genotypes, while some *ospC* major groups including T and U have been associated with localized skin infection [14], [18], [19]. Consistent with the *ospC* studies, isolates within CC37, associated with localized skin infection, belonged to either *ospC* major groups T or U, all but one isolate found in CC4, associated with disseminated infection, belonged to either *ospC* major groups K or H and all isolates within CC7 and CC16 that were also associated with disseminated infection were identified as members of *ospC* major groups B and I, respectively. In addition, isolates that belonged to a single *ospC* major group (e.g. B, I, K or U), but exhibited different dissemination properties, were further divided among distinct clonal complexes or clonal complexes and singletons. These differences could be explained by horizontal gene transfer and recombination at *ospC* that over time would result in the dissociation of *ospC* major groups from genes which contribute to the ability to disseminate. A number of studies provide genetic evidence of recombination and horizontal gene transfer at *ospC* [7], [8], [13], [26], [28], [51]. While the mechanisms underlying these processes in nature are unknown, mixed infections in the tick or host, that would be required for genetic exchange, have been documented [10], [43], [52]–[57].

It has been suggested that the *B. burgdorferi* population structure is dominated by frequency dependent selection acting at *ospC* [10]–[12]. Based on this, and the strong linkage disequilibrium observed between *ospC* and other genomic markers, it has been proposed that *ospC* is a lineage-defining gene [28]. While at a certain scale a strong association between *ospC* and various chromosomal and/or plasmid-encoded loci [5], [6], [8], [10], [13] has been shown, this linkage proved not to be absolute at a wider spatial scale [8], [42], [58]. The present results, based on concatenated sequences of eight housekeeping genes suggest that *B. burgdorferi* is not subdivided in a manner that corresponds to *ospC* major groups. First, almost all *ospC* major groups were associated with multiple STs. Second, although most STs were comprised predominantly of isolates with the same *ospC* major group, related STs were assembled into clonal complexes that did not reflect a simple clonal descent of *ospC* major group within *B. burgdorferi* lineages. Taken together, the data indicate that while *ospC* major group may be a relatively stable characteristic of a ST, the use of *ospC* typing as a means of strain identification for epidemiological, ecological and population genetic studies is not reliable.

Patients from New York and Wisconsin were infected with two distinct, but genetically and phylogenetically closely related, populations of *B. burgdorferi*. Only two STs were identified in patients from both locations. The presence of identical *B. burgdorferi* STs in *I. scapularis* ticks from the Northeast and Upper Midwest of North America have been previously explained as either a reminiscence of once overlapping populations of *B. burgdorferi* or the result of limited gene flow [41]–[43]. Geographical structuring of *B. burgdorferi* in Lyme disease patients from New York and Wisconsin captured by MLST in this study was similar to that previously reported in *I. scapularis* questing ticks from the Northeast and Upper Midwest of North America [42], [43]. The mirroring geographic patterns of *B. burgdorferi* in humans and *I. scapularis* ticks are not surprising given that infection is acquired peri-domestically by *I. scapularis* tick bites [59], [60]. In contrast to MLST, and in concordance with other studies, *ospC* did not provide a clear signal of geographical structuring of *B. burgdorferi* [23], [41], [42], [54]. While there was a significantly different frequency distribution of *ospC* major groups between patients from New York and those from Wisconsin, the vast majority of *ospC* major groups infected patients in both regions. It is conceivable that due to evolution of *ospC* in ancestral spirochete populations and balancing selection [10]–[12], [27], [61], [62] geographic structuring of *ospC* major groups may be more obscure than that observed at MLST loci. Nevertheless, a large-scale geospatial analysis of *ospC* sequences from *I. scapularis* ticks demonstrated that despite a substantial overlap of *ospC* types in northeastern and midwestern populations, differences in frequency distribution allowed a subdivision at the longitude of 83°W [8]. Given the differences in clinical outcomes associated with specific genotypes, the spatial differences of *B. burgdorferi* observed at the MLST and *ospC* levels and its epidemiological implications warrant further investigation.

The MLST analysis performed in this study suggests that recombination was likely to generate some of the *B. burgdorferi* diversity at the MLST loci. For example, ST52 strains possess a *clpA* allele that differs from its ancestral *clpA* allele at seven nucleotide positions and is shared by two STs that are members of different clonal complexes. It is very unlikely that seven independent point mutations would accumulate at a single locus while no changes would be present on the other seven loci, and that two unrelated STs would share alleles that arose by accumulation of seven point mutations. It can, therefore, be assumed that this allele arose through recombination rather than point mutation [63]. Indeed, genetic evidence of recombination has been recently reported for the *B. burgdorferi* chromosome suggesting that apart from mutation and genetic drift, recombination may also have some impact on the diversification of *B. burgdorferi* [28], [64]. While it is known that recombination may obscure phylogenetic signals, the definition of clonal complexes via goeBURST on the basis of allele identity (an integer) rather than sequence diversity [65] tends to yield discrete clusters of related organisms that appear to be stable over decades, if not centuries, even at medium levels of homologous recombination [66], [67].

In conclusion, MLST analysis of *B. burgdorferi* strains isolated from Lyme disease patients suggests the existence of *B. burgdorferi* lineages with differential pathogenic properties in humans. The data indicate that MLST strain identification is better suited for epidemiological, ecological and population genetics studies than is *ospC* typing and suggest that MLST provides finer resolution of differentially pathogenic *B. burgdorferi* strains than does *ospC* typing. The results further demonstrate that humans in New York and Wisconsin are infected with two separate, but genetically related, populations of *B. burgdorferi*. While the data provide new and important insights into the population structure of *B. burgdorferi*, a much larger collection of diverse isolates must be examined to better understand the full extent of *B. burgdorferi* strain diversity and population structure in Lyme disease patients, and relationships between human disease, STs and clonal complexes.

## Materials and Methods

### Ethics Statement

All human subjects from New York were enrolled in prospective studies approved by the Institutional Review Board (IRB) of New York Medical College for which they had provided written informed consent at the Lyme Disease Diagnostic Center between 1991 and 2006. Samples and data from Wisconsin patients were collected in the past as a part of routine care/diagnostic testing and were accessed under approved IRB protocols of the Marshfield Clinic Research Foundation. A waiver of informed consent has been granted by the IRB. All data were analyzed anonymously.

### Clinical Isolates

A total of 146 *B. burgdorferi* clinical isolates recovered from 146 Lyme disease patients (i.e., a single isolate per patient) from New York and Wisconsin (states located in the northeastern and upper Midwestern regions of US, respectively) were analyzed in this study. Seventy six isolates were cultured from erythema migrans lesions (n = 60) or blood (n = 16) of patients diagnosed at the Lyme Disease Diagnostic Center at New York Medical College in Valhalla, NY between 1991 and 2005, and 70 isolates were recovered from erythema migrans skin lesions (n = 65) or cerebrospinal fluid specimens (n = 5) of patients diagnosed at the Marshfield Clinic in Wisconsin between 1993 and 2003. Specimens were collected and cultured as described elsewhere [18], [68]–[70].

The New York isolates analyzed in this study belong to a large collection of more than 400 clinical isolates that had been previously typed at the *rrs-rrl*A IGS and *ospC* loci [13], [18], [19]. Therefore, to better cover the full diversity of *B. burgdorferi* genotypes found in the collection, 3 to 7 isolates per *ospC* major group were selected for this study. For those *ospC* major groups that consisted of isolates from skin and blood, representatives from both were included. No preselection was made for the Wisconsin isolates.

Patients included in this study were diagnosed with early Lyme disease and were classified as having either localized or disseminated infection. Localized infection was defined by a single culture positive erythema migrans skin lesion in the absence of a positive blood culture or clinical and/or microbiological evidence of dissemination to a second site. Disseminated infection was defined by a positive blood or cerebrospinal fluid culture, multiple erythema migrans lesions and/or neurological findings.

### DNA Extraction and MLST

DNA from low-passage (passages 1 to 5) *B. burgdorferi* was isolated from the clinical samples with either the IsoQuick kit (Orca Research, Bothell, WA) or the Gentra PureGene DNA Isolation Kit (Qiagen Inc., Valencia, CA). Eight housekeeping loci (*clpA*, *clpX*, *nifS*, *pepX*, *pyrG*, *recG*, *rplB*, and *uvrA*) were amplified by nested PCR and sequenced using PCR primers in both directions (Genewiz, Inc., South Plainfield, NJ) as described previously [7]. Quality control of DNA traces was conducted manually in DNASTAR (Lasergene plc, USA). Isolates that produced ambiguous sequence results were cloned by limiting dilution and sequencing of all eight loci were performed on two clones from each isolate. Samples that still produced ambiguous sequence results were considered mixed and removed from further analysis. Sequences of individual genes were compared to each other and to sequences in the MLST database. Existing sequences were assigned allelic numbers and new sequences were automatically assigned consecutive allele numbers by the MLST database. New consecutive sequence type (ST) numbers were assigned to allelic profiles with novel combinations. All alleles and STs are accessible at the MLST website (http://www.borrelia.mlst.net/) hosted at Imperial College London (London, UK). STs of 23 clinical isolates used in this study have been previously reported [7].

### *ospC* Analysis

To determine the *ospC* genotypes of Wisconsin clinical isolates, the *ospC* gene was amplified by PCR using OC6 (+) and OC623 (−) primers as described previously [10] and the PCR products were sequenced in both directions (Genewiz, Inc., South Plainfield, NJ). The sequence quality control and further analysis of unambiguous sequence results were performed as described above. To determine the major *ospC* groups, the *ospC* sequences were compared to existing sequences of major *ospC* groups found worldwide, according to the previously defined criteria [12].

### Sequence Alignment

For each unique ST the sequences of eight housekeeping loci were concatenated to produce an in-frame sequence of 4,785 bp. Multiple sequence alignment was generated with the ClustalW algorithm and BioEdit software [71] by using default parameters, followed by manual inspection. The alignment was made on the translated amino acid sequences and then back-translated to nucleotide sequences to ensure in-frame alignment.

### Phylogenetic Analysis

Maximum likelihood (ML) and maximum parsimony (MP) trees were constructed using PhyML 3.0 [72] and MEGA 5.05 [73], respectively. To construct the ML tree, the optimal maximum likelihood model of nucleotide substitution was determined using jModeltest [74], a neighbor-joining starting tree, nearest-neighborhood interchange and subtree pruning and regrafting. Approximate likelihood ratios (aLRT) were calculated using the Shimodaira-Hasegawa (SH)-like procedure. Rate heterogeneity among sites was examined assuming a discrete gamma distribution with eight rate categories. For the MP tree only parsimony informative sites were used, with 20 replicates of random taxa addition, and close-neighbor-interchange branch swapping. Support for internal nodes was estimated by the nonparametric bootstrap method, with 1,000 replications under a maximum parsimony criterion. Clonal complexes (CCs) were identified using the goeBURST algorithm implemented in the Phyloviz software [75] and consisted of STs that shared 6 of 8 alleles with at least one other ST in the complex. Major and minor clonal complexes were defined as groups of three or more STs and two STs, respectively. Singletons did not belong to any clonal complex, as they differed from every other ST in the data set at three or more of the eight MLST loci. The goeBURST and phylogenetic tree analyses included STs identified in this study and all previously published STs from the northeastern and upper midwestern US and adjoining areas of Canada [7], [41]–[43]. The *I. scapularis* nymphs and adults included in the analyses performed in this study were collected off vegetation, pets and humans in the northeastern and upper midwestern US and adjoining areas of Canada between 2004–2008 [41]–[43]. We did not attempt to compare the diversity and/or frequency distribution of STs in Lyme disease patients to that found in questing *I. scapularis* ticks because: *(i)* there was no temporal overlap between tick and patient samples, *(ii)* ticks were collected from much wider geographic areas than clinical samples, and *(iii)* clinical samples from New York were preselected based on their *ospC* major group.

### Statistical Analysis

To test whether there are significant differences between the allelic profiles of *B. burgdorferi* from the two geographic locations (New York and Wisconsin) a permutation test was performed as described previously [7]. Correspondence analysis in STATA SE 11.0 (Statacorp LP, College Station, TX) was used to assess whether the STs, clonal complexes or *ospC* major groups were significantly (P<0.05) associated with an outcome of localized or disseminated infection. The correspondence analysis of clonal complexes was conducted in three ways using: *(i)* isolates that were members of major and minor clonal complexes, and isolates that were classified as singletons (each singleton was considered a separate clonal complex), *(ii)* isolates that were members of major and minor clonal complexes only, and *(iii)* isolates that belonged to major clonal complexes only. To compare the ability of clonal complexes versus *ospC* major groups to predict disseminated or localized disease, these three analyses were repeated with *ospC* major groups as the explanatory variables. Correspondence analysis was also used to compare the frequency distribution of *ospC* major groups from 291 skin isolates obtained from New York patients during 1991–2005 and published previously [13], [19] to that from 65 skin isolates obtained from Wisconsin patients and analyzed in the present study. No pre-selection based on *ospC* typing was performed for this set of data. The level of significance was P<0.05 throughout.

## Supporting Information

Figure S1 **Results of correspondence analysis using only isolates belonging to major or minor clonal complexes.** The x-axis indicates the coordinates of the individual data points. Coordinates for all localized and disseminated infection cases are indicated by the filled diamond and filled square respectively. The strength of association of individual clonal complexes (CCs) (graph A) and *ospC* major groups (graph B) with disseminated or localized infection is demonstrated by the position of unfilled triangles on the x-axis relative to the filled square and lozenge, respectively. The degree of influence of individual clonal complexes or *ospC* major groups in the correspondence analysis (their contribution to the total inertia) is shown by their position along the y-axis. Identities of the particularly influential clonal complexes and *ospC* major groups are indicated.
(TIF)

Table S1 **Properties of** ***B. burgdorferi*** **samples.**
(DOC)

Table S2 **Results of correspondence analysis assessing differences in frequency of** ***ospC*** **major groups in skin samples of Lyme disease patients from New York and Wisconsin.**
(DOC)

## References

[1] Dennis DE, Hayes EB (2002) In: Gary SJ, Kahl O, Lane RS, Stanek G, editors. Lyme borreliosis: Biology, Epidemiology and Control. CABI publishing, Oxford, UK. 251–280.

[2] Center of Disease Control and Prevention (2012) Lyme Disease - United States, Available: http://www.cdc.gov/lyme/stats/index.html.Accessed 14 March 2013.

[3] Wormser GP, Dattwyler RJ, Shapiro ED, Halperin JJ, Steere AC, et al (2006) The clinical assessment, treatment, and prevention of Lyme disease, human granulocytic anaplasmosis, and babesiosis: clinical practice guidelines by the Infectious Diseases Society of America. Clin Infect Dis 43: 1089–1134. 17029130 10.1086/508667

[4] Wormser GP (2006) Hematogenous dissemination in early Lyme disease. Wien Klin Wochenschr 118: 634–637. 17160600 10.1007/s00508-006-0688-9

[5] Attie O, Bruno JF, Xu Y, Qiu D, Luft BJ, et al (2007) Co-evolution of the outer surface protein C gene (*ospC*) and intraspecific lineages of *Borrelia burgdorferi* sensu stricto in the northeastern United States. Infect Genet Evol 7: 1–12. 16684623 10.1016/j.meegid.2006.02.008

[6] Bunikis J, Garpmo U, Tsao J, Berglund J, Fish D, et al (2004) Sequence typing reveals extensive strain diversity of the Lyme borreliosis agents *Borrelia burgdorferi* in North America and *Borrelia afzelii* in Europe. Microbiology 150: 1741–1755. 15184561 10.1099/mic.0.26944-0

[7] Margos G, Gatewood AG, Aanensen DM, Hanincova K, Terekhova D, et al (2008) MLST of housekeeping genes captures geographic population structure and suggests a European origin of *Borrelia burgdorferi* . Proc Natl Acad Sci U S A 105: 8730–8735. 18574151 10.1073/pnas.0800323105 PMC2435589

[8] Barbour AG, Travinsky B (2010) Evolution and distribution of the *ospC* gene, a transferable serotype determinant of *Borrelia burgdorferi* . MBio 1: e00153. 20877579 10.1128/mBio.00153-10 PMC2945197

[9] Liveris D, Wormser GP, Nowakowski J, Nadelman R, Bittker S, et al (1996) Molecular typing of *Borrelia burgdorferi* from Lyme disease patients by PCR-restriction fragment length polymorphism analysis. J Clin Microbiol 34: 1306–1309. 8727927 10.1128/jcm.34.5.1306-1309.1996 PMC229006

[10] Qiu WG, Dykhuizen DE, Acosta MS, Luft BJ (2002) Geographic uniformity of the Lyme disease spirochete (*Borrelia burgdorferi*) and its shared history with tick vector (*Ixodes scapularis*) in the Northeastern United States. Genetics 160: 833–849. 11901105 10.1093/genetics/160.3.833 PMC1462027

[11] Qiu WG, Bosler EM, Campbell JR, Ugine GD, Wang IN, et al (1997) A population genetic study of *Borrelia burgdorferi* sensu stricto from eastern Long Island, New York, suggested frequency-dependent selection, gene flow and host adaptation. Hereditas 127: 203–216. 9474903 10.1111/j.1601-5223.1997.00203.x

[12] Wang IN, Dykhuizen DE, Qiu W, Dunn JJ, Bosler EM, et al (1999) Genetic diversity of *ospC* in a local population of *Borrelia burgdorferi* sensu stricto. Genetics 151: 15–30. 9872945 10.1093/genetics/151.1.15 PMC1460459

[13] Hanincova K, Liveris D, Sandigursky S, Wormser GP, Schwartz I (2008) *Borrelia burgdorferi* sensu stricto is clonal in patients with early Lyme borreliosis. Appl Environ Microbiol 74: 5008–5014. 18539816 10.1128/AEM.00479-08 PMC2519259

[14] Seinost G, Dykhuizen DE, Dattwyler RJ, Golde WT, Dunn JJ, et al (1999) Four clones of *Borrelia burgdorferi* sensu stricto cause invasive infection in humans. Infect Immun 67: 3518–3524. 10377134 10.1128/iai.67.7.3518-3524.1999 PMC116539

[15] Brisson D, Dykhuizen DE (2004) *ospC* diversity in *Borrelia burgdorferi*: different hosts are different niches. Genetics 168: 713–722. 15514047 10.1534/genetics.104.028738 PMC1448846

[16] Jones KL, Glickstein LJ, Damle N, Sikand VK, McHugh G, et al (2006) *Borrelia burgdorferi* genetic markers and disseminated disease in patients with early Lyme disease. J Clin Microbiol 44: 4407–4413. 17035489 10.1128/JCM.01077-06 PMC1698394

[17] Hanincova K, Kurtenbach K, Diuk-Wasser M, Brei B, Fish D (2006) Epidemic spread of Lyme borreliosis, northeastern United States. Emerg Infect Dis 12: 604–611. 16704808 10.3201/eid1204.051016 PMC3294694

[18] Wormser GP, Liveris D, Nowakowski J, Nadelman RB, Cavaliere LF, et al (1999) Association of specific subtypes of *Borrelia burgdorferi* with hematogenous dissemination in early Lyme disease. J Infect Dis 180: 720–725. 10438360 10.1086/314922

[19] Wormser GP, Brisson D, Liveris D, Hanincova K, Sandigursky S, et al (2008) *Borrelia burgdorferi* genotype predicts the capacity for hematogenous dissemination during early Lyme disease. J Infect Dis 198: 1358–1364. 18781866 10.1086/592279 PMC2776734

[20] Dykhuizen DE, Brisson D, Sandigursky S, Wormser GP, Nowakowski J, et al (2008) The propensity of different *Borrelia burgdorferi* sensu stricto genotypes to cause disseminated infections in humans. Am J Trop Med Hyg 78: 806–810. 18458317 PMC2387051

[21] Strle K, Jones KL, Drouin EE, Li X, Steere AC (2011) *Borrelia burgdorferi* RST1 (OspC type A) genotype is associated with greater inflammation and more severe Lyme disease. Am J Pathol 178: 2726–2739. 21641395 10.1016/j.ajpath.2011.02.018 PMC3123987

[22] Earnhart CG, Buckles EL, Dumler JS, Marconi RT (2005) Demonstration of OspC type diversity in invasive human Lyme disease isolates and identification of previously uncharacterized epitopes that define the specificity of the OspC murine antibody response. Infect Immun 73: 7869–7877. 16299277 10.1128/IAI.73.12.7869-7877.2005 PMC1307023

[23] Brisson D, Vandermause MF, Meece JK, Reed KD, Dykhuizen DE (2010) Evolution of northeastern and midwestern *Borrelia burgdorferi*, United States. Emerg Infect Dis 16: 911–917. 20507740 10.3201/eid1606.090329 PMC3086229

[24] Spratt BG, Maiden MC (1999) Bacterial population genetics, evolution and epidemiology. Philos Trans R Soc Lond B Biol Sci 354: 701–710. 10365396 10.1098/rstb.1999.0423 PMC1692550

[25] Tilly K, Krum JG, Bestor A, Jewett MW, Grimm D, et al (2006) *Borrelia burgdorferi* OspC protein required exclusively in a crucial early stage of mammalian infection. Infect Immun 74: 3554–3564. 16714588 10.1128/IAI.01950-05 PMC1479285

[26] Wang G, van Dam AP, Dankert J (1999) Evidence for frequent OspC gene transfer between *Borrelia valaisiana* sp. nov. and other Lyme disease spirochetes. FEMS Microbiol Lett 177: 289–296. 10474195 10.1111/j.1574-6968.1999.tb13745.x

[27] Dykhuizen DE, Baranton G (2001) The implications of a low rate of horizontal transfer in *Borrelia* . Trends Microbiol 9: 344–350. 11435109 10.1016/s0966-842x(01)02066-2

[28] Haven J, Vargas LC, Mongodin EF, Xue V, Hernandez Y, et al (2011) Pervasive recombination and sympatric genome diversification driven by frequency-dependent selection in *Borrelia burgdorferi*, the Lyme disease bacterium. Genetics 189: 951–966. 21890743 10.1534/genetics.111.130773 PMC3213364

[29] Maiden MC, Bygraves JA, Feil E, Morelli G, Russell JE, et al (1998) Multilocus sequence typing: a portable approach to the identification of clones within populations of pathogenic microorganisms. Proc Natl Acad Sci U S A 95: 3140–3145. 9501229 10.1073/pnas.95.6.3140 PMC19708

[30] Maiden MC (2006) Multilocus sequence typing of bacteria. Annu Rev Microbiol 60: 561–588. 16774461 10.1146/annurev.micro.59.030804.121325

[31] Jolley KA, Kalmusova J, Feil EJ, Gupta S, Musilek M, et al (2000) Carried meningococci in the Czech Republic: a diverse recombining population. J Clin Microbiol 38: 4492–4498. 11101585 10.1128/jcm.38.12.4492-4498.2000 PMC87626

[32] Enright MC, Spratt BG (1998) A multilocus sequence typing scheme for *Streptococcus pneumoniae*: identification of clones associated with serious invasive disease. Microbiology 144: 3049–3060. 9846740 10.1099/00221287-144-11-3049

[33] Zhou J, Enright MC, Spratt BG (2000) Identification of the major Spanish clones of penicillin-resistant pneumococci via the Internet using multilocus sequence typing. J Clin Microbiol 38: 977–986. 10698983 10.1128/jcm.38.3.977-986.2000 PMC86318

[34] Enright MC, Day NP, Davies CE, Peacock SJ, Spratt BG (2000) Multilocus sequence typing for characterization of methicillin-resistant and methicillin-susceptible clones of *Staphylococcus aureus* . J Clin Microbiol 38: 1008–1015. 10698988 10.1128/jcm.38.3.1008-1015.2000 PMC86325

[35] Enright MC, Robinson DA, Randle G, Feil EJ, Grundmann H, et al (2002) The evolutionary history of methicillin-resistant *Staphylococcus aureus* (MRSA). Proc Natl Acad Sci U S A 99: 7687–7692. 12032344 10.1073/pnas.122108599 PMC124322

[36] Feil EJ, Cooper JE, Grundmann H, Robinson DA, Enright MC, et al (2003) How clonal is *Staphylococcus aureus*? J Bacteriol 185: 3307–3316. 12754228 10.1128/JB.185.11.3307-3316.2003 PMC155367

[37] Dingle KE, Colles FM, Wareing DR, Ure R, Fox AJ, et al (2001) Multilocus sequence typing system for *Campylobacter jejuni* . J Clin Microbiol 39: 14–23. 11136741 10.1128/JCM.39.1.14-23.2001 PMC87672

[38] Schouls LM, Reulen S, Duim B, Wagenaar JA, Willems RJ, et al (2003) Comparative genotyping of *Campylobacter jejuni* by amplified fragment length polymorphism, multilocus sequence typing, and short repeat sequencing: strain diversity, host range, and recombination. J Clin Microbiol 41: 15–26. 12517820 10.1128/JCM.41.1.15-26.2003 PMC149617

[39] Wareing DR, Ure R, Colles FM, Bolton FJ, Fox AJ, et al (2003) Reference isolates for the clonal complexes of *Campylobacter jejuni* . Lett Appl Microbiol 36: 106–110. 12535131 10.1046/j.1472-765x.2003.01270.x

[40] Arvand M, Feil EJ, Giladi M, Boulouis HJ, Viezens J (2007) Multi-locus sequence typing of *Bartonella henselae* isolates from three continents reveals hypervirulent and feline-associated clones. PLoS One 2: e1346. 18094753 10.1371/journal.pone.0001346 PMC2147075

[41] Ogden NH, Margos G, Aanensen DM, Drebot MA, Feil EJ, et al (2011) Investigation of genotypes of *Borrelia burgdorferi* in *Ixodes scapularis* ticks collected during surveillance in Canada. Appl Environ Microbiol 77: 3244–3254. 21421790 10.1128/AEM.02636-10 PMC3126474

[42] Margos G, Tsao JI, Castillo-Ramirez S, Girard YA, Hamer SA, et al (2012) Two boundaries separate *Borrelia burgdorferi* populations in North America. Appl Environ Microbiol 78: 6059–6067. 22729536 10.1128/AEM.00231-12 PMC3416618

[43] Hoen AG, Margos G, Bent SJ, Diuk-Wasser MA, Barbour AG, et al (2009) Phylogeography of *Borrelia burgdorferi* in the eastern United States reflects multiple independent Lyme disease emergence events. Proc Natl Acad Sci U S A 106: 15013–15018. 19706476 10.1073/pnas.0903810106 PMC2727481

[44] Margos G, Vollmer SA, Cornet M, Garnier M, Fingerle V, et al (2009) A new *Borrelia* species defined by multilocus sequence analysis of housekeeping genes. Appl Environ Microbiol 75: 5410–5416. 19542332 10.1128/AEM.00116-09 PMC2725479

[45] Nadelman RB, Hanincova K, Mukherjee P, Liveris D, Nowakowski J, et al (2012) Differentiation of reinfection from relapse in recurrent Lyme disease. N Engl J Med 367: 1883–1890. 23150958 10.1056/NEJMoa1114362 PMC3526003

[46] Terekhova D, Iyer R, Wormser GP, Schwartz I (2006) Comparative genome hybridization reveals substantial variation among clinical isolates of *Borrelia burgdorferi* sensu stricto with different pathogenic properties. J Bacteriol 188: 6124–6134. 16923879 10.1128/JB.00459-06 PMC1595389

[47] Brisson D, Baxamusa N, Schwartz I, Wormser GP (2011) Biodiversity of *Borrelia burgdorferi* strains in tissues of Lyme disease patients. PLoS One 6: e22926. 21829670 10.1371/journal.pone.0022926 PMC3150399

[48] Wang G, Ojaimi C, Iyer R, Saksenberg V, McClain SA, et al (2001) Impact of genotypic variation of *Borrelia burgdorferi* sensu stricto on kinetics of dissemination and severity of disease in C3H/HeJ mice. Infect Immun 69: 4303–4312. 11401967 10.1128/IAI.69.7.4303-4312.2001 PMC98500

[49] Wang G, Ojaimi C, Wu H, Saksenberg V, Iyer R, et al (2002) Disease severity in a murine model of Lyme borreliosis is associated with the genotype of the infecting *Borrelia burgdorferi* sensu stricto strain. J Infect Dis 186: 782–791. 12198612 10.1086/343043 PMC2773673

[50] Earnhart CG, Leblanc DV, Alix KE, Desrosiers DC, Radolf JD, et al (2010) Identification of residues within ligand-binding domain 1 (LBD1) of the *Borrelia burgdorferi* OspC protein required for function in the mammalian environment. Mol Microbiol 76: 393–408. 20199597 10.1111/j.1365-2958.2010.07103.x PMC2917209

[51] Lin T, Oliver JH Jr, Gao L (2002) Genetic diversity of the outer surface protein C gene of southern *Borrelia* isolates and its possible epidemiological, clinical, and pathogenetic implications. J Clin Microbiol 40: 2572–2583. 12089279 10.1128/JCM.40.7.2572-2583.2002 PMC120588

[52] Bunikis J, Tsao J, Luke CJ, Luna MG, Fish D, et al (2004) *Borrelia burgdorferi* infection in a natural population of *Peromyscus leucopus* mice: a longitudinal study in an area where Lyme Borreliosis is highly endemic. J Infect Dis 189: 1515–1523. 15073690 10.1086/382594

[53] Swanson KI, Norris DE (2008) Presence of multiple variants of *Borrelia burgdorferi* in the natural reservoir *Peromyscus leucopus* throughout a transmission season. Vector Borne Zoonotic Dis 8: 397–405. 18399776 10.1089/vbz.2007.0222 PMC2978052

[54] Travinsky B, Bunikis J, Barbour AG (2010) Geographic differences in genetic locus linkages for *Borrelia burgdorferi* . Emerg Infect Dis 16: 1147–1150. 20587192 10.3201/eid1607.091452 PMC3321895

[55] Crowder CD, Matthews HE, Schutzer S, Rounds MA, Luft BJ, et al (2010) Genotypic Variation and Mixtures of Lyme Borrelia in *Ixodes* Ticks from North America and Europe. PLoS One 5: e10650. 20498837 10.1371/journal.pone.0010650 PMC2871049

[56] Guttman DS, Wang PW, Wang IN, Bosler EM, Luft BJ, et al (1996) Multiple infections of *Ixodes scapularis* ticks by *Borrelia burgdorferi* as revealed by single-strand conformation polymorphism analysis. J Clin Microbiol 34: 652–656. 8904432 10.1128/jcm.34.3.652-656.1996 PMC228864

[57] Hamer SA, Hickling GJ, Sidge JL, Rosen ME, Walker ED, et al (2011) Diverse *Borrelia burgdorferi* strains in a bird-tick cryptic cycle. Appl Environ Microbiol 77: 1999–2007. 21257811 10.1128/AEM.02479-10 PMC3067335

[58] Qiu WG, Bruno JF, McCaig WD, Xu Y, Livey I, et al (2008) Wide distribution of a high-virulence *Borrelia burgdorferi* clone in Europe and North America. Emerg Infect Dis 14: 1097–1104. 18598631 10.3201/eid1407.070880 PMC2600328

[59] Maupin GO, Fish D, Zultowsky J, Campos EG, Piesman J (1991) Landscape Ecology of Lyme Disease in a Residential Area of Westchester County, New York. Am J Epidemiol 133. 10.1093/oxfordjournals.aje.a115823 2035514

[60] Falco C, Fish D (1988) Ticks Parasitizing Humans in a Lyme Disease Endemic Area of Southern New York State. Am J Epidemiol 128: 1146–1152. 3189288 10.1093/oxfordjournals.aje.a115057

[61] Qiu WG, Schutzer SE, Bruno JF, Attie O, Xu Y, et al (2004) Genetic exchange and plasmid transfers in *Borrelia burgdorferi* sensu stricto revealed by three-way genome comparisons and multilocus sequence typing. Proc Natl Acad Sci U S A 101: 14150–14155. 15375210 10.1073/pnas.0402745101 PMC521097

[62] Kurtenbach K, Gatewood A, Bent SJ, Vollmer SA, Ogden NH, et al.. (2010) Population Biology of Lyme Borreliosis Spirochetes. In: Robinson DA, Falush D, Feil EJ, editors. Bacterial Population Genetics in Infectious Disease. 217–245.

[63] Feil EJ, Smith JM, Enright MC, Spratt BG (2000) Estimating recombinational parameters in *Streptococcus pneumoniae* from multilocus sequence typing data. Genetics 154: 1439–1450. 10747043 10.1093/genetics/154.4.1439 PMC1461021

[64] Vitorino LR, Margos G, Feil EJ, Collares-Pereira M, Ze-Ze L, et al (2008) Fine-scale phylogeographic structure of *Borrelia lusitaniae* revealed by multilocus sequence typing. PLoS One 3: e4002. 19104655 10.1371/journal.pone.0004002 PMC2602731

[65] Feil EJ, Li BC, Aanensen DM, Hanage WP, Spratt BG (2004) eBURST: inferring patterns of evolutionary descent among clusters of related bacterial genotypes from multilocus sequence typing data. J Bacteriol 186: 1518–1530. 14973027 10.1128/JB.186.5.1518-1530.2004 PMC344416

[66] Turner KM, Hanage WP, Fraser C, Connor TR, Spratt BG (2007) Assessing the reliability of eBURST using simulated populations with known ancestry. BMC Microbiol 7: 30. 17430587 10.1186/1471-2180-7-30 PMC1865383

[67] Feil EJ, Holmes EC, Bessen DE, Chan MS, Day NP, et al (2001) Recombination within natural populations of pathogenic bacteria: short-term empirical estimates and long-term phylogenetic consequences. Proc Natl Acad Sci U S A 98: 182–187. 11136255 10.1073/pnas.98.1.182 PMC14565

[68] Wang G, Iyer R, Bittker S, Cooper D, Small J, et al (2004) Variations in Barbour-Stoenner-Kelly culture medium modulate infectivity and pathogenicity of *Borrelia burgdorferi* clinical isolates. Infect Immun 72: 6702–6706. 15501807 10.1128/IAI.72.11.6702-6706.2004 PMC523011

[69] Schwartz I, Wormser GP, Schwartz JJ, Cooper D, Weissensee P, et al (1992) Diagnosis of early Lyme disease by polymerase chain reaction amplification and culture of skin biopsies from erythema migrans lesions. J Clin Microbiol 30: 3082–3088. 1452688 10.1128/jcm.30.12.3082-3088.1992 PMC270592

[70] Mitchell PD, Reed KD, Vandermause MF, Melski JW (1993) Isolation of *Borrelia burgdorferi* from skin biopsy specimens of patients with erythema migrans. American journal of clinical pathology 1: 104–107. 10.1093/ajcp/99.1.104 8422007

[71] Hall TA (1999) BioEdit: a user friendly biological sequence alignment editor an analysis program for Window 95/98/NT. Nucleic Acids Symp Ser 41: 95–98.

[72] Guindon S, Dufayard JF, Lefort V, Anisimova M, Hordijk W, et al (2010) New Algorithms and Methods to Estimate Maximum-Likelihood Phylogenies: Assessing the Performance of PhyML 3.0. Systematic Biology 59: 307–321. 20525638 10.1093/sysbio/syq010

[73] Tamura K, Peterson D, Peterson N, Stecher G, Nei M, et al (2011) MEGA5: molecular evolutionary genetics analysis using maximum likelihood, evolutionary distance, and maximum parsimony methods. Mol Biol Evol 28: 2731–2739. 21546353 10.1093/molbev/msr121 PMC3203626

[74] Posada D (2008) jModelTest: phylogenetic model averaging. Mol Biol Evol 25: 1253–1256. 18397919 10.1093/molbev/msn083

[75] Francisco AP, Bugalho M, Ramirez M, Carrico JA (2009) Global optimal eBURST analysis of multilocus typing data using a graphic matroid approach. BMC Bioinformatics 10: 152. 19450271 10.1186/1471-2105-10-152 PMC2705362

