# Supplementary figures and images for: Multilocus Sequence Typing of Borrelia burgdorferi Suggests Existence of Lineages with Differential Pathogenic Properties in Humans
Source: PLoS One. 2013 Sep 17;8(9):e73066. doi: 10.1371/journal.pone.0073066 (PMC3775742; doi:10.1371/journal.pone.0073066)

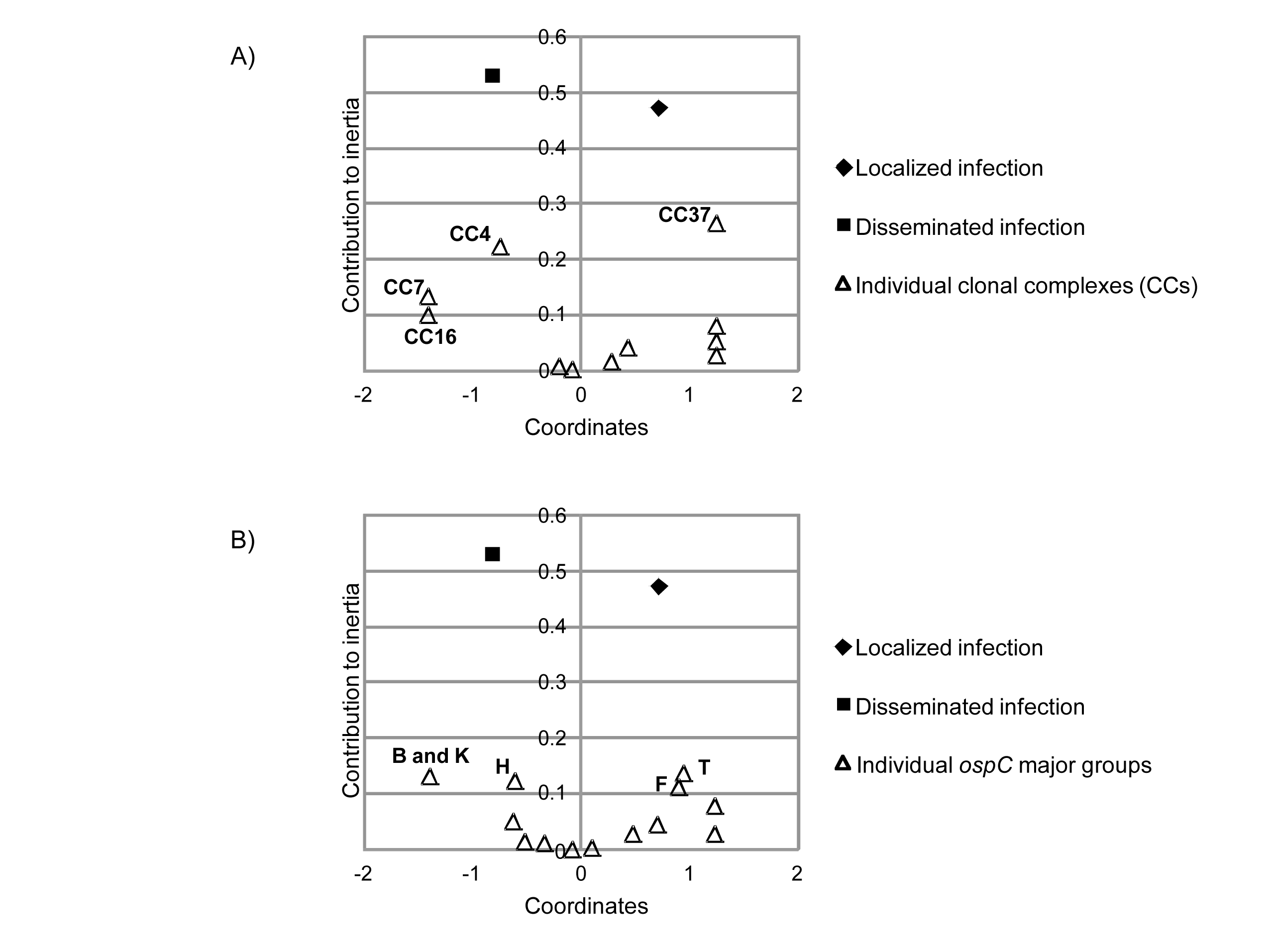

Supplement: Figure S1 — Results of correspondence analysis using only isolates belonging to major or minor clonal complexes. The x-axis indicates the coordinates of the individual data points. Coordinates for all localized and disseminated infection cases are indicated by the filled diamond and filled square respectively. The strength of association of individual clonal complexes (CCs) (graph A) and ospC major groups (graph B) with disseminated or localized infection is demonstrated by the position of unfilled triangles on the x-axis relative to the filled square and lozenge, respectively. The degree of influence of individual clonal complexes or ospC major groups in the correspondence analysis (their contribution to the total inertia) is shown by their position along the y-axis. Identities of the particularly influential clonal complexes and ospC major groups are indicated. (TIF) [file pone.0073066.s001.tif]
