# Supplementary material for: Multilocus Sequence Typing of Borrelia burgdorferi Suggests Existence of Lineages with Differential Pathogenic Properties in Humans
Source: PLoS One. 2013 Sep 17;8(9):e73066. doi: 10.1371/journal.pone.0073066 (PMC3775742; doi:10.1371/journal.pone.0073066)
Supplement: Table S1 — Properties of B. burgdorferi samples. (DOC) [file pone.0073066.s002.doc]

Table S1. Properties of *B. burgdorferi* samples.

| **Strain** | **ST** | ***ospC* Group^a^** | ***ospC* allele^b^** | **Source** | **Year** | **Type of Infection**^c^ | **Geographical Region** |
| --- | --- | --- | --- | --- | --- | --- | --- |
| **B491** | 1 | A | *ospC* AT1 | Skin | 2000 | Disseminated | New York |
| **B515** | 1 | A | *ospC* AT1 | Skin | 2000 | Localized | New York |
| **BL203** | 1 | A | *ospC* AT1 | Blood | 1997 | Disseminated | New York |
| **BL206** | 1 | A | *ospC* AT1 | Blood | 1997 | Disseminated | New York |
| **BL268** | 1 | A | *ospC* AT1 | Blood | 1999 | Disseminated | New York |
| **B390** | 3 | K | *ospC* AT11 | Skin | 1998 | ND | New York |
| **B435** | 3 | K | *ospC* AT11 | Skin | 1999 | Disseminated | New York |
| **B483** | 3 | N | *ospC* AT13 | Skin | 2000 | ND | New York |
| **B504** | 3 | K | *ospC* AT11 | Skin | 2000 | Disseminated | New York |
| **BL224** | 3 | K | *ospC* AT11 | Blood | 1997 | Disseminated | New York |
| **BL608** | 3 | K | *ospC* AT11 | Blood | 2004 | Disseminated | New York |
| **MC104** | 4 | H | *ospC* AT8 | CSF | 1998 | Disseminated | Wisconsin |
| **B395** | 4 | H | *ospC* AT8 | Skin | 1998 | Localized | New York |
| **B471** | 4 | H | *ospC* AT8 | Skin | 2000 | Disseminated | New York |
| **B509** | 4 | H | *ospC* AT8 | Skin | 2000 | Disseminated | New York |
| **BL546** | 4 | H | *ospC* AT8 | Blood | 2002 | Disseminated | New York |
| **BL631** | 4 | H | *ospC* AT8 | Blood | 2005 | Disseminated | New York |
| **MR641** | 4 | H | *ospC* AT8 | Skin | 2002 | Disseminated | New York |
| **MR708** | 4 | H | *ospC* AT8 | Skin | 2005 | Disseminated | New York |
| **B365** | 7 | B | *ospC* AT2 | Skin | 1997 | Disseminated | New York |
| **B373** | 7 | B | *ospC* AT2 | Skin | 1997 | Disseminated | New York |
| **B416** | 7 | B | *ospC* AT2 | Skin | 1998 | ND | New York |
| **MR623** | 7 | B | *ospC* AT2 | Skin | 2001 | Disseminated | New York |
| **B156** | 8 | F | *ospC* AT6 | Skin | 1994 | ND | New York |
| **B267** | 8 | F | *ospC* AT6 | Skin | 1995 | Localized | New York |
| **B353** | 8 | F | *ospC* AT6 | Skin | 1997 | Localized | New York |
| **B488** | 8 | F | *ospC* AT6 | Skin | 2000 | Localized | New York |
| **Strain** | **ST** | ***ospC* Group** | ***ospC* allele**^b^ | **Source** | **Year** | **Type of Infection**^c^ | **Geographical Region** |
| **B516** | 8 | F | *ospC* AT6 | Skin | 2000 | ND | New York |
| **BL597** | 8 | F | *ospC* AT6 | Blood | 2003 | Disseminated | New York |
| **MR627** | 8 | F | *ospC* AT6 | Skin | 2001 | Localized | New York |
| **MR628** | 9 | N | *ospC* AT13 | Skin | 2001 | Disseminated | New York |
| **MR654** | 9 | N | *ospC* AT13 | Skin |  | Disseminated | New York |
| **MR661** | 9 | N | *ospC* AT13 | Skin | 2002 | Disseminated | New York |
| **BL515** | 11 | C | *ospC* AT3 | Blood | 2001 | Disseminated | New York |
| **BL538** | 11 | C | *ospC* AT3 | Blood | 2001 | Disseminated | New York |
| **MR635** | 11 | C | *ospC* AT3 | Skin | 2001 | Disseminated | New York |
| **MC96** | 12 | M | **M2** | Skin | 1997 | Disseminated | Wisconsin |
| **B356** | 12 | M | *ospC* AT12 | Skin | 1997 | Disseminated | New York |
| **BL522** | 12 | M | *ospC* AT12 | Blood | 2001 | Disseminated | New York |
| **MR659** | 12 | M | *ospC* AT12 | Skin | 2002 | Localized | New York |
| **MR670** | 12 | M | *ospC* AT12 | Skin | 2003 | Localized | New York |
| **MR704** | 12 | M | *ospC* AT12 | Skin | 2005 | Localized | New York |
| **MR716** | 12 | M | *ospC* AT12 | Skin | 2005 | Disseminated | New York |
| **MR726** | 12 | M | *ospC* AT12 | Skin | 2005 | Localized | New York |
| **B268** | 14 | G | *ospC* AT7 | Skin | 1995 | Localized | New York |
| **B327** | 14 | G | *ospC* AT7 | Skin | 1996 | ND | New York |
| **B369** | 14 | G | *ospC* AT7 | Skin | 1997 | Localized | New York |
| **B385** | 14 | G | *ospC* AT7 | Skin | 1998 | Localized | New York |
| **BL630** | 14 | G | *ospC* AT7 | Blood | 2005 | Disseminated | New York |
| **MR616** | 14 | G | *ospC* AT7 | Skin | 2001 | Localized | New York |
| **B500** | 15 | I | *ospC* AT9 | Skin | 2000 | Disseminated | New York |
| **B331** | 16 | I | *ospC* AT9 | Skin | 1996 | ND | New York |
| **B361** | 16 | I | *ospC* AT9 | Skin | 1997 | Disseminated | New York |
| **B408** | 16 | I | *ospC* AT9 | Skin | 1998 | ND | New York |
| **B143** | 18 | U | *ospC* AT15 | Skin | 1994 | Localized | New York |
| **Strain** | **ST** | **ospC Group** | ***ospC* allele**^b^ | **Source** | **Year** | **Type of Infection**^c^ | **Geographical Region** |
| **B399** | 18 | U | *ospC* AT15 | Skin | 1998 | Localized | New York |
| **B485** | 18 | U | *ospC* AT15 | Skin | 2000 | Localized | New York |
| **MR607** | 18 | U | *ospC* AT15 | Skin | 2001 | Localized | New York |
| **MR662** | 18 | U | *ospC* AT15 | Skin | 2002 | Localized | New York |
| **MR676** | 18 | U | *ospC* AT15 | Skin | 2003 | Localized | New York |
| **B348** | 19 | E | *ospC* AT5 | Skin | 1997 | Disseminated | New York |
| **B351** | 19 | E | *ospC* AT5 | Skin | 1997 | Localized | New York |
| **B397** | 19 | E | *ospC* AT5 | Skin | 1998 | Localized | New York |
| **B418** | 19 | E | *ospC* AT5 | Skin | 1998 | Disseminated | New York |
| **CDC146** | 19 | E | *ospC* AT5 | Skin | 1991 | Localized | New York |
| **MR640** | 19 | E | *ospC* AT5 | Skin | 2002 | Disseminated | New York |
| **MC111** | 29 | L | L | CSF | 1998 | Disseminated | Wisconsin |
| **MC115** | 29 | L | **L1** | Skin | 1999 | ND | Wisconsin |
| **MC123** | 29 | L | **L1** | Skin | 1999 | ND | Wisconsin |
| **MC125** | 29 | L | **L1** | Skin | 2000 | Disseminated | Wisconsin |
| **MC89** | 29 | L | L | Skin | 1997 | Localized | Wisconsin |
| **MC93** | 29 | L | L | Skin | 1997 | Disseminated | Wisconsin |
| **MC108** | 30 | B | *ospC* AT2 | Skin | 1998 | Localized | Wisconsin |
| **MC132** | 30 | B | *ospC* AT2 | Skin | 2001 | Disseminated | Wisconsin |
| **MC105** | 32 | H | Hb | Skin | 1998 | Localized | Wisconsin |
| **MC107** | 32 | H | Hb | Skin | 1998 | Localized | Wisconsin |
| **MC112** | 32 | H | Hb | Skin | 1999 | Disseminated | Wisconsin |
| **MC113** | 32 | H | Hb | Skin | 1999 | Disseminated | Wisconsin |
| **MC130** | 32 | H | Hb | Skin | 2000 | Disseminated | Wisconsin |
| **MC139** | 32 | H | Hb | Skin | 2001 | Disseminated | Wisconsin |
| **MC143** | 32 | H | Hb | Skin | 2002 | Localized | Wisconsin |
| **MC119** | 32 | H | Hb | Skin | 1999 | Localized | Wisconsin |
| **B236** | 34 | J | *ospC* AT10 | Skin | 1995 | Localized | New York |
| **Strain** | **ST** | **ospC Group** | ***ospC* allele**^b^ | **Source** | **Year** | **Type of Infection**^c^ | **Geographical Region** |
| **BL285** | 34 | J | *ospC* AT10 | Blood | 1999 | Disseminated | New York |
| **CDC161** | 34 | J | *ospC* AT10 | Skin | 1991 | Disseminated | New York |
| **BL131** | 35 | I | *ospC* AT9 | Blood | 1994 | Disseminated | New York |
| **BL324** | 36 | N | *ospC* AT13 | Blood | 2000 | Disseminated | New York |
| **MR730** | 37 | T | *ospC* AT14 | Skin | 2005 | Localized | New York |
| **MR731** | 37 | T | *ospC* AT14 | Skin | 2005 | Localized | New York |
| **B240** | 38 | D | *ospC* AT4 | Skin | 1995 | ND | New York |
| **B477** | 38 | D | *ospC* AT4 | Skin | 2000 | Localized | New York |
| **B75** | 38 | D | *ospC* AT4 | Skin | 1993 | Disseminated | New York |
| **MR692** | 40 | E3 | *ospC* AT16 | Skin | 2003 | Localized | New York |
| **BL325** | 42 | B | *ospC* AT2 | Blood | 2000 | Disseminated | New York |
| **MC82** | 43 | N | *ospC* AT13 | Skin | 1996 | Localized | Wisconsin |
| **MC90** | 47 | U | *ospC* AT15 | Skin | 1997 | Localized | Wisconsin |
| **MC88** | 48 | G | *ospC* AT7 | Skin | 1996 | ND | Wisconsin |
| **MC91** | 48 | G | *ospC* AT7 | Skin | 1997 | Localized | Wisconsin |
| **MC97** | 48 | G | *ospC* AT7 | Skin | 1997 | Disseminated | Wisconsin |
| **MC122** | 51 | D | Db | Skin | 1999 | Localized | Wisconsin |
| **MC131** | 51 | D | Db | Skin | 2001 | Localized | Wisconsin |
| **MC85** | 52 | E3 | E3 | Skin | 1996 | Localized | Wisconsin |
| **MC121** | 55 | A | *ospC* AT1 | CSF | 1999 | Disseminated | Wisconsin |
| **MC133** | 55 | A | *ospC* AT1 | CSF | 2001 | Disseminated | Wisconsin |
| **MC98** | 55 | A | *ospC* AT1 | Skin | 1998 | Localized | Wisconsin |
| **MC134** | 55 | C | Cb | Skin | NA | ND | Wisconsin |
| **MC73** | 56 | F | Fc | Skin | 1995 | ND | Wisconsin |
| **MC78** | 56 | F | Fc | Skin | 1996 | Localized | Wisconsin |
| **MC149** | 221 | O | Oa | Skin | 2002 | Localized | Wisconsin |
| **MC79** | 221 | O | Oa | Skin | 1996 | Disseminated | Wisconsin |
| **MC95** | 221 | O | Oa | Skin | 1997 | Disseminated | Wisconsin |
| **Strain** | **ST** | **ospC Group** | ***ospC* allele**^b^ | **Source** | **Year** | **Type of Infection**^c^ | **Geographical Region** |
| **MC83** | 222 | U | *ospC* AT15 | Skin | 1996 | Disseminated | Wisconsin |
| **MC128** | 222 | U | *ospC* AT15 | Skin | 2000 | Localized | Wisconsin |
| **MC127** | 223 | T | **T1** | Skin | 2000 | Localized | Wisconsin |
| **MC145** | 223 | T | **T1** | Skin | 2002 | Localized | Wisconsin |
| **MC74** | 224 | H | Hb | Skin | 1995 | Localized | Wisconsin |
| **MC75** | 224 | H | Hb | Skin | 1995 | Disseminated | Wisconsin |
| **MC77** | 224 | H | Hb | Skin | 1996 | Disseminated | Wisconsin |
| **MC80** | 224 | H | Hb | Skin | 1996 | Disseminated | Wisconsin |
| **MC110** | 225 | F3 | F3 | Skin | 1998 | Localized | Wisconsin |
| **MC120** | 225 | F3 | F3 | Skin | 1999 | Localized | Wisconsin |
| **MC135** | 226 | D | Db | Skin | 2001 | Localized | Wisconsin |
| **MC114** | 227 | I | *ospC* AT9 | Skin | 1999 | Localized | Wisconsin |
| **MC129** | 228 | I | *ospC* AT9 | Skin | 2000 | Localized | Wisconsin |
| **MC101** | 229 | E | **E1** | Skin | 1998 | Localized | Wisconsin |
| **MC92** | 229 | E | **E1** | Skin | 1997 | ND | Wisconsin |
| **MC84** | 230 | A | *ospC* AT1 | Skin | 1996 | ND | Wisconsin |
| **MC150** | 231 | F | Fc | Skin | 2003 | Localized | Wisconsin |
| **MC117** | 232 | B3 | B3 | Skin | 1999 | Localized | Wisconsin |
| **MC118** | 232 | B3 | B3 | Skin | 1999 | ND | Wisconsin |
| **MC144** | 233 | B3 | B3 | Skin | 2002 | Localized | Wisconsin |
| **MC146** | 233 | B3 | B3 | Skin | 2002 | Localized | Wisconsin |
| **MC126** | 234 | K | *ospC* AT11 | Skin | 2000 | Localized | Wisconsin |
| **MC76** | 234 | K | *ospC* AT11 | Skin | 1996 | ND | Wisconsin |
| **MC81** | 234 | K | *ospC* AT11 | Skin | 1996 | Localized | Wisconsin |
| **MC86** | 234 | K | *ospC* AT11 | CSF | 1996 | Disseminated | Wisconsin |
| **MC99** | 234 | K | *ospC* AT11 | Skin | 1998 | Localized | Wisconsin |
| **MC109** | 235 | X | **X1** | Skin | 1998 | Localized | Wisconsin |
| **MC103** | 236 | Y | Y | Skin | 1998 | Disseminated | Wisconsin |
| **Strain** | **ST** | **ospC Group** | ***ospC* allele**^b^ | **Source** | **Year** | **Type of Infection**^c^ | **Geographical Region** |
| **MC100** | 237 | J | *ospC* AT10 | Skin | 1998 | Localized | Wisconsin |
| **MC102** | 302 | F | *ospC* AT8 | Skin | 1998 | Localized | Wisconsin |
| **MC106** | 409 | M | **M2** | Skin | 1998 | ND | Wisconsin |
| **MC137** | 410 | N | *ospC* AT13 | Skin | 2001 | Disseminated | Wisconsin |
| **MC138** | 411 | N | *ospC* AT13 | Skin | 2001 | Localized | Wisconsin |
| **MC147** | 412 | D | Db | Skin | 2002 | Localized | Wisconsin |
| **MC148** | 413 | C | Cb | Skin | 2002 | Localized | Wisconsin |

*^a^* *ospC* major groups designation according to [8,12]. The GenBank accession numbers of reference sequences are EU482041 to EU482051 for *ospC* A to K, EU375832 for *ospC* L, EU482052 and EU482053 for *ospC* M and N, EU482054 and EU482055 for *ospC* T and U, EF592542 for *ospC* B3, EU482056 for *ospC* E3, and EF592547 for *ospC* F3. *ospC* major groups X and Y were not published at the time this article was written but were available in GenBank under accession numbers HM047876 and HM047875, respectively.

*^b^* *ospC* allele names are based on [8,13]; alleles newly identified in this study are presented in bold and were deposited in GenBank with the accession numbers KC682474 to KC682478.

*^c^* A case of disseminated infection was defined by one or more of the following conditions *(i)* positive blood culture, *(ii)* multiple erythema migrans lesions, and *(iii)* neurological findings. A case of localized infection was defined by a single culture positive erythema migrans skin lesion.

ST, sequence type; ND, not determined; CSF, cerebrospinal fluid.
