# Supplementary material for: Multilocus Sequence Typing of Borrelia burgdorferi Suggests Existence of Lineages with Differential Pathogenic Properties in Humans
Source: PLoS One. 2013 Sep 17;8(9):e73066. doi: 10.1371/journal.pone.0073066 (PMC3775742; doi:10.1371/journal.pone.0073066)
Supplement: Table S2 — Results of correspondence analysis assessing differences in frequency of ospC major groups in skin samples of Lyme disease patients from New York and Wisconsin. (DOC) [file pone.0073066.s003.doc]

Table S2. Results of correspondence analysis assessing differences in frequency of *ospC* major groups in skin samples of patients from New York and Wisconsin.

| **Categories** | **Coordinates** | **Contribution to inertia** |
| --- | --- | --- |
| **Region** | | |
| **Wisconsin** | 1.613 | 0.818 |
| **New York** | -0.359 | 0.182 |
| ***ospC* major group*^a^*** | | |
| **A** | -0.478 | 0.053 |
| **B** | -0.445 | 0.037 |
| **B3** | **2.784** | **0.15** |
| **C** | 1.082 | 0.023 |
| **D** | 1.082 | 0.045 |
| **E** | -0.194 | 0.003 |
| **E3** | 1.082 | 0.011 |
| **F** | 0.428 | 0.011 |
| **F3** | **2.784** | **0.075** |
| **G** | -0.052 | 0 |
| **H** | **1.014** | **0.124** |
| **I** | -0.31 | 0.01 |
| **J** | 0.231 | 0.001 |
| **K** | **-0.469** | **0.095** |
| **L** | **2.784** | **0.187** |
| **M** | -0.096 | 0.001 |
| **N** | -0.109 | 0.001 |
| **O** | **1.933** | **0.072** |
| **T** | 1.082 | 0.023 |
| **U** | 0.11 | 0.001 |
| **X** | 2.784 | 0.037 |
| **Y** | 2.784 | 0.037 |

Overall: Total inertia = 0.336, χ^2^ = 119.88, P < 0.001, N = 357*^b^*

*^a^* Frequencies of *ospC* major groups that were particularly influential (i.e. contributed more to the overall inertia and significance of the model) are in bold. Greater frequencies of *ospC* major groups in Wisconsin were responsible for the overall significance of the model, as indicated by the coordinates (5 *ospC* major groups were more frequent in Wisconsin while only one was more frequent in New York).
